# Supplementary material for: 8q24 genetic variation and comprehensive haplotypes altering familial risk of prostate cancer
Source: Nat Commun. 2020 Mar 23;11:1523. doi: 10.1038/s41467-020-15122-1 (PMC7089954; doi:10.1038/s41467-020-15122-1)
Supplement: Supplementary file 2 — Supplementary Information [file 41467_2020_15122_MOESM2_ESM.pdf]

Supplementary Information

8q24 genetic variation and comprehensive haplotypes altering familial risk of prostate cancer

Dupont et al.

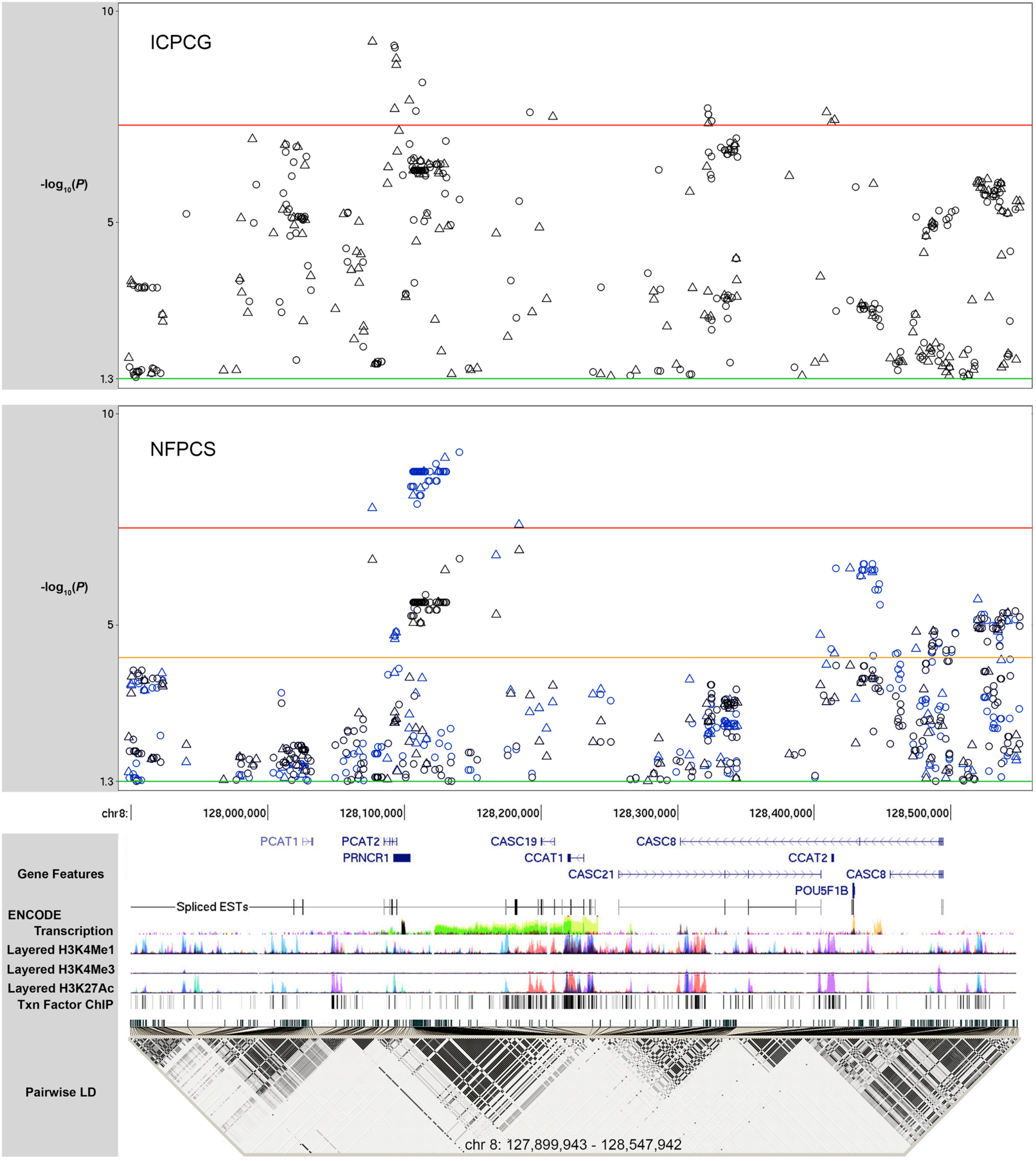

ICPCG
NFPCS
-log10(P)
10
5
1.3
chr8:
128,000,000
128,100,000
128,200,000
128,300,000
128,400,000
128,500,000
Gene Features
PCAT1
PCAT2
PRNCR1
CASC19
CCAT1
CASC21
CASC8
CCAT2
CASC8
POU5F1B
Spliced ESTs
ENCODE Transcription
Layered H3K4Me1
Layered H3K4Me3
Layered H3K27Ac
Txn Factor ChIP
Pairwise LD
chr 8: 127,899,943 - 128,547,942

Supplementary Figure 1 Association of 433 genetic variants across the 8q24 interval with hereditary prostate cancer (HPC). At top is a Manhattan plot of statistical significance within a comparison of ICPCG HPC cases with controls, depicting variant positions on the x-axis and $-\log_{10} P$ values on the y-axis. The horizontal red line corresponds to the genome-wide significance threshold of $P = 5 \times 10^{-8}$ to accommodate multiple comparisons; the green line to $P = 0.05$. Each data point depicts the result of a multiplicative logistic regression model (additive genetic model), with two-sided significance assessed using Wald tests. Triangle data points depict genotyped and circles depict imputed variants. The middle Manhattan plot illustrates the results of analogous tests of these variants in the NFPCS. Data point color indicates comparison of either of two NFPCS case groups to controls: black depicts HPC cases, while blue depicts an expanded case group that additionally includes FPC cases. The yellow line corresponds to $P = 7 \times 10^{-5}$ (Bonferroni correction for 765 variants tested in the NFPCS). All plotted variants were concordantly associated with HPC in the two independent study populations at a nominal $P \leq 0.05$ level. At bottom is a corresponding genomic map and pairwise LD matrix, depicting R^2 values among HPC cases of the combined studies. Genomic features are drawn from the UCSC Genome Browser for the hg19 assembly. Truncation of the lower aspect of the LD matrix omits depiction of existing LD between distant variant pairs.

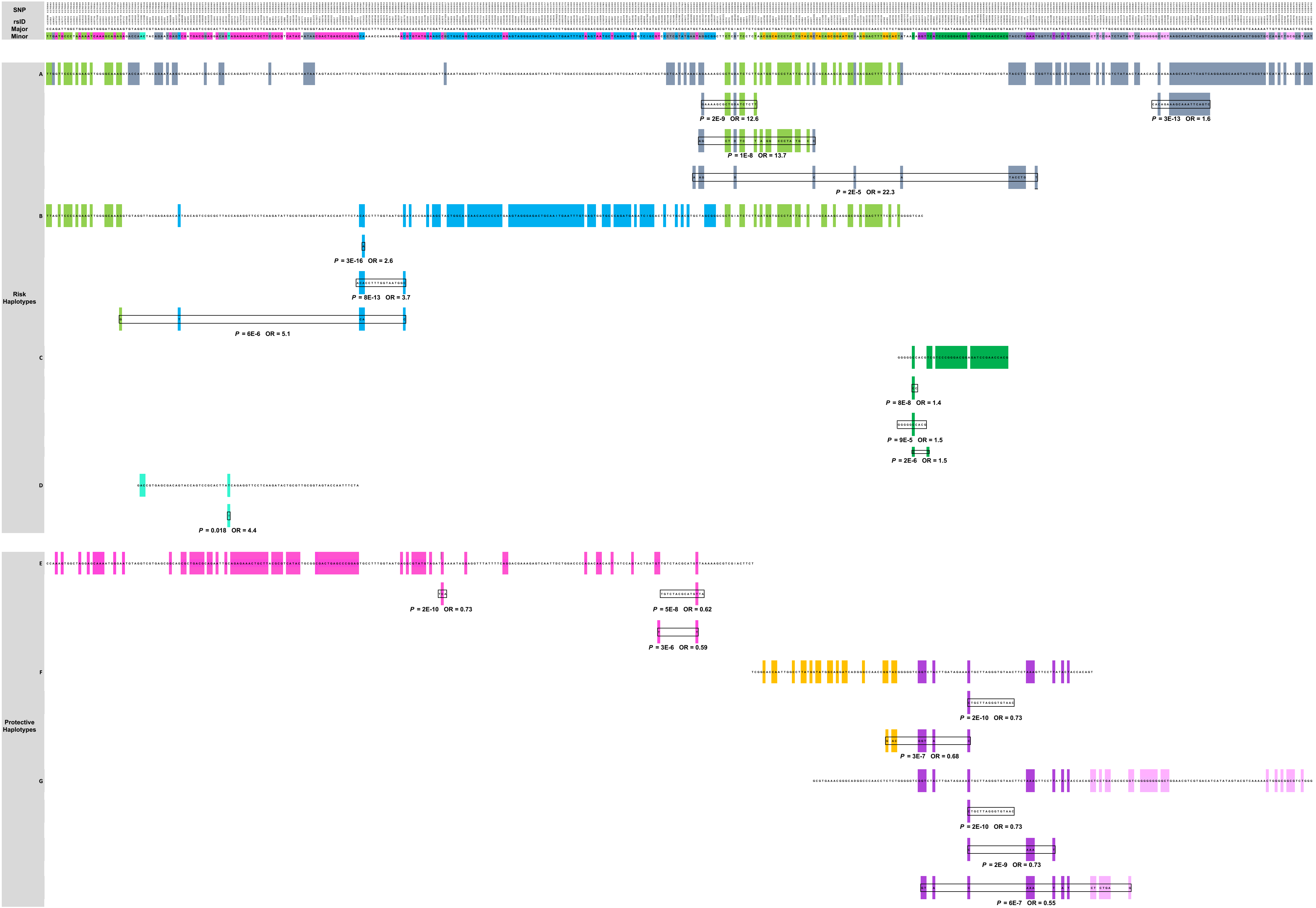
SNP
rsID
Major
Minor
A
P = 2E-9 OR = 12.6
P = 3E-13 OR = 1.6
P = 1E-8 OR = 13.7
P = 2E-5 OR = 22.3
B
P = 3E-16 OR = 2.6
P = 8E-13 OR = 3.7
P = 6E-6 OR = 5.1
Risk Haplotypes
C
P = 8E-8 OR = 1.4
P = 9E-5 OR = 1.5
P = 2E-6 OR = 1.5
D
P = 0.018 OR = 4.4
E
P = 2E-10 OR = 0.73
P = 5E-8 OR = 0.62
P = 3E-6 OR = 0.59
F
P = 2E-10 OR = 0.73
P = 3E-7 OR = 0.68
Protective Haplotypes
G
P = 2E-10 OR = 0.73
P = 2E-9 OR = 0.73
P = 6E-7 OR = 0.55

Supplementary Figure 2. Risk-altering haplotypes. Each of the 433 concordantly significant variants is designated at top (chromosome, position, rsID, major and minor allele). Four risk and three protective haplotypes are illustrated, with color coding of variant minor alleles that distinguish a given haplotype from the other risk-altering haplotypes (shading as described for **Figure 3**). Under sliding-window analysis evaluating from one (single allele) to 20 adjacent variants (haplotypes), the windows of peak significance and effect size are denoted for each corresponding haplotype. For some, the window of peak significance and the window of peak effect is the same. A secondary sliding-window analysis was also done for each haplotype, constraining the variants evaluated to the subset distinguishing it from the other risk-altering haplotypes. For example, the window of peak effect size for haplotype A spanned 19 adjacent variants with $P = 2 \times 10^{-9}$, OR = 12.6. Upon omitting variants with minor alleles that did not distinguish haplotype A from the remaining risk-altering haplotypes, the window of peak effect spanned 14 variants with $P = 2 \times 10^{-5}$, OR = 22.3. Alleles in light green shade are shared with haplotype B and could also contribute to the effect of haplotype A. If included in the analysis, then the window of peak effect spans 20 variants, $P = 1 \times 10^{-8}$, OR = 13.7. Tests of association between hapotype and disease used multiplicative logistic regression models (additive genetic models), with two-sided significance assessed using Wald tests.

University of Washington Center for Mendelian Genomics (UW-CMG)
Joshua D. Smith[1], Suzanne M. Leal[2], Peter Anderson[1], Tamara J. Bacus[1], Kati J. Buckingham[1], Colleen P. Davis[1], Christopher Frazar[1], Danielle Giroux[1], William W. Gordon[1], Martha Horike-Pyne[1], Jameson R. Hurless[1], Gail P. Jarvik[1], Eric Johanson[1], Tom Kolar[1], Melissa P. MacMillan[1], Colby T. Marvin[1], Sean McGee[1], Daniel J. McGoldrick[1], Betselote Mekonnen[1], Patrick M. Nielsen[1], Karynne Patterson[1], Ben Pelle[1], Aparna Radhakrishnan[1], Matthew A. Richardson[1], Peggy D. Robertson[1], Erica L. Ryke[1], Aimee M. Schantz[1], Isabelle Schrauwen[2], Kathryn M. Shively[1], Monica Tackett[1], Machiko S. Threlkeld[1], Marc Wegener[1], Jeffrey M. Weiss[1], Marsha M. Wheeler[1], Janson J. White[1], Qian Yi[1], Di Zhang[2], and Xiaohong Zhang[1]

[1]University of Washington
[2]Columbia University
