# Supplementary material for: 8q24 genetic variation and comprehensive haplotypes altering familial risk of prostate cancer
Source: Nat Commun. 2020 Mar 23;11:1523. doi: 10.1038/s41467-020-15122-1 (PMC7089954; doi:10.1038/s41467-020-15122-1)
Supplement: Supplementary file 3 — Description of Additional Supplementary Files [file 41467_2020_15122_MOESM3_ESM.pdf]

Description of Additional Supplementary Files

File Name: Supplementary Data 1
Description: Supplementary results of association analyses. First tab: Results of tests of association with HPC for 765 nominally-significant 8q24 variants in ICPCG data, evidence of replication in NFPCS, and evaluation within the combined subjects. A likelihood ratio chi-squared test was employed for rare variants (P values marked by an asterisk). P values of variants for which there were no case or no control carriers are marked with symbols † or ‡, respectively. Where there were no NFPCS carriers the P value field holds the § symbol. False discovery rate (FDR Q values) were evaluated in NFPCS subjects.53 An evaluation of NFPCS association with adjustment for age is provided. Ages of ICPCG controls were not recorded. An evaluation of association among combined subjects under adjustment for the first four principal components of genetic ancestry is also presented. Second tab: Results of tests of association with either HPC or FPC (both combined). Third tab: Results of tests of association with HPC without and with adjustment for all previously known 8q24 GWAS prostate cancer risk variants for men of European ancestry (from www.ebi.ac.uk/gwas/home, Hoffmann et. al. (ref 33), or Matejcic et. al. (ref 34)). Minor alleles are colorized to indicate haplotype marked as in Figures 2 and 3.

File Name: Supplementary Data 2
Description: Mutation detection of a high-risk haplotype A. The table presents variants detected by sequencing seven NFPCS cases, with corresponding population allele frequencies from gnomAD and TOPMed. Phased diplotypes are presented for each case in a region sharing haplotype A (grey shade). Genotypes are unphased for a given case in a recombinant flank that does not share haplotype A. Where the allele observed for a variant is annotated in gnomAD, we employ gnomAD allele nomenclature to facilitate comparison. Each of the mutation candidates is designated by green highlight on the second tab. Eight of eleven identified mutation candidates (second tab) were previously unknown. Additional tabs provide detail for each mutational candidate. The final tab presents a translocation variant (insertion of 3q13 sequence in inverted orientation into 8q24).

File Name: Supplementary Data 3
Description: Analyses of sentinel variants detected by the RISSc algorithm, and by Matejcic et al.26.
